# Resurrected microorganisms: a plethora of resting bacteria underway for human interaction

**DOI:** 10.1186/s13568-024-01750-z

**Published:** 2024-09-28

**Authors:** Arshia Amin, Inam Ullah Khan, Mehroze Amin, Maliha Fatima, Wasim Sajjad, Tawaf Ali Shah, Turki M. Dawoud, Gezahign Fentahun Wondmie

**Affiliations:** 1 Faculty of Health and Life Sciences, Capital University of Science and Technology, Islamabad, Pakistan; 2 Gomal University, Dera Ismail Khan, Pakistan; 3 School of Biological Sciences, University of Punjab, Lahore, Pakistan; 4 Department of Biological Sciences, National University of Medical Sciences, Rawalpindi, 46000 Pakistan; 5 College of agriculture engineering and food science, Shandong University of Technology, Zibo, 255000 China; 6 Department of Botany and Microbiology, College of Science, King Saud University, P. O. BOX 2455, 11451 Riyadh, Saudi Arabia; 7 Department of Biology, Bahir Dar University, P.O.Box 79, Bahir Dar, Ethiopia

**Keywords:** Extremophiles, Glaciers, Metagenome, Microbial community, Proteobacteria, Freshwater resources, Ecological significance

## Abstract

Glaciers, which form due to the accumulation of snow, play a crucial role in providing freshwater resources, supporting river systems, and maintaining ecosystem stability. Pakistan is habitat to over 5000 glaciers, primarily located in the Hindukash, Himalaya, and Karakoram mountain ranges. Understanding the microbial communities thriving in these extreme environments becomes of utmost importance. These glaciers offer a unique perspective on extremophile adaptation, as they harbor microorganisms that are capable of surviving and thriving under harsh conditions. Glacial melting poses a significant threat to ancient microbiomes, potentially leading to the resurgence of epidemics and exposure of life to paleomicrobiota. Mostly glacial microbiome is evenly distributed and shows similar diversity. With the threat of resurrection of ages old microbiome and its incorporation into the waters have raised a major concern for revival of epidemics and exposure of life with paleanmicrobiota again. This has led the scientist to deeply observe the bacterial flora embedded in the cryonite holes of glaciers. This study aims to investigate the bacterial diversity within various glaciers of Pakistan using metagenomic techniques. Kamri, Burzil, Siachin, Baltoro, Shigar Basin, Biafo and Panama Glaciers designated from G1 to G7 respectively were chosen from Pakistan. Through rigorous physicochemical analyses, distinct characteristics among glaciers are revealed, including variations in temperature, depth, electrical conductivity, pH levels, and nutrient concentrations. The exploration of alpha diversity, employing metrics such as Chao1, Shannon, Simpson, and Inverse Simpson indices, offers valuable insights into the richness, evenness, and dominance of species within different samples. Beta diversity was calculated by using R software. The vegan package was used for NMSD, cluster and PCoA analysis based on Bray–Curtis distance. PCA analysis was done by using prcomp package from R software. Based on OTU abundance and environmental factor data, DCA analysis was done to determine the linear model from the gradient value (RDA) and the unimodal model (CCA). results were compiled by drawing cluster dendrogram which predicts the patterns of similarity and dissimilarity between different samples. Notably, phyla *Proteobacteria* emerge as the dominant phylum, accompanied by *Actinobacteria*, *Firmicutes*, and *Bacteroidetes*. The dendrogram shows five clusters, with close similarity between G1 and G4, glacier samples G3 and G8, and G2 and G7. Seasonal variations in glacier physicochemical properties were also observed, with summer samples having shallower depths, lower temperatures, and slightly acidic pH. In contrast, winter samples have higher electrical conductivity and sulfur content. Ultimately, this research provides a foundational framework for comprehending glacier ecosystems, their resident microbial communities, and their broader ecological significance. The study highlights the potential public health risks linked to the release of ancient microorganisms due to climate change, emphasizing the need for comprehensive monitoring and research to mitigate potential public health threats.

**Supplementary Information:**

The online version contains supplementary material available at 10.1186/s13568-024-01750-z.

## Introduction

Accumulation of snow year by year results in the formation of glaciers that contain microorganisms depending upon the climate (Ambrosini et al. 2017). More than 5000 glaciers reported are from Pakistan, the largest glaciers are in Hindukush, Himalaya, and Karakoram mountains (Williams et al. 2010). The glaciers are major drinking water supply basins, and their daily melting of ice nourishes about sixty large and small country rivers. Such reservoirs preserve and store water that is important for development and economy. Glaciers cover 10% of the earth’s atmosphere and about 80–85% of the earth’s temperature is less than five degrees Celsius that includes the Alps, seas, polar regions, mesosphere, and stratosphere (Cowan et al. 2010). Some of these low-temperature ecosystems are deep sea and oceans with temperatures ranging from − 1 to 4 °C accompanied by ecosystems like cold deserts, lakes, caves, sea ice and glaciers. One of the most common climates on Earth is a low-temperature climate that includes the polar zone, high mountain glaciers in Europe, 95% of the oceans, the upper atmosphere, and artificial environments like refrigerators and freezers, all maintain temperatures below five degrees Celsius (Margesin and Miteva 2011). This shows that low temperature is the most common extreme condition that is widely dispersed on Earth. Glaciers occur in areas where snow formation supports climatic and topographic conditions (Gomes and Steiner 2004).

Glaciers have an extremely cold habitat in which every type of microbes cannot survive, only a few can be viable that are tolerant against the low temperature and thrive in harsh conditions. All the microbes that grow at temperatures less than 20 °C are called psychrophilic microbes. Psychrophilic organisms can survive below 20 °C temperature however species that grow at higher optimal temperatures and withstand low temperatures are called psycrhotolerant (Margesin and Miteva 2011). In recent years, the terms e*urypsychrophiles* and *stenopsychrophils* have been proposed as more suitable for describing the temperature range that an organism can withstand (Rothschild and Mancinelli 2001).

Psychrophiles have the ability to cope with low pH, high concentration of metals, low availability of nutrients, and no light (Rafiq 2016). In glaciers, bacteria are present on a large scale, in which only 1% of the bacteria are culturable in the environment and 99% are unculturable due to lack of suitable natural environment conditions (Anesio and Laybourn-Parry 2012). The cryoconite holes formed by little debris particles in the glaciers have an important key role in the most biologically active environment and adaptation to the cold (Kohshima 1984).

Microbes play a significant role in subglacial weathering and make availability for minerals and other nutrients for lifeforms which is significant in climate change (Montross et al. 2013). The previous study shows that microbial communities include bacteria, archaea, fungi, and viruses also (Boetius et al. 2015). Few microbial active organisms were identified in the extreme environment, with glaciers having confined habitats and are highly selective which makes the environment favorable for adaptation and speciation (Garcia-Lopez et al. 2019). Glacier ice is a special habitat that chronologically retains microorganisms and it is mostly present in Greenland and Antarctica, which equate to about 10% of Earth’s terrestrial surface and hold 77% of the planet’s freshwater (Segawa et al. 2010b).

While microorganisms had long been present in glacier ice and other frozen habitats, the interest in ice microbiology was not revived until Abyzov’s groundbreaking research on the deep ice over Lake Vostok in Antarctica in the late 1980s. These authors used microscopy, cultivation and 14 °C coded substrates to analyze ice core samples from the surface to 3000 m deep and found viable cells of different sizes and shapes at relatively low concentrations (Segawa et al. 2010a). Additionally, for the first time the variations in cell numbers were related to the content of mineral particles and temperature shifts with a higher number of dust particles and cells that occur during colder periods. Later, a systematic analysis conducted on glacier ice core samples from different geographic locations, ranging from 5 to 20,000 years of age, showed that various bacteria can be recovered with success (Miteva 2008). This Glacial microflora is made up of 90% by bacteria and 10% by fungi and archaea. Research about glaciers from several parts of the world such as Alaska (Xiang et al. 2009a, b), China (Cheng and Foght 2007), Canada (Christner et al. 2000), and America (Zhang et al. 2010) proposed that glacier ice core samples have Protobacteria as the most dominant bacterial phyla, which account for approximately 65% of the total bacterial community. Within the Proteobacteria, the beta-proteobacteria are the dominant class. The dominant bacterial community in glacier ice cores is composed of Gram-positive, non-spore-forming Actinobacteria and Firmicutes taxa, along with significant numbers of Gram-negative bacteria from Proteobacteria and Bacteroidetes phyla, successfully recovered and cultured across different locations (Handelsman et al. 1998). Species of these phyla were consistent with colored and viscous colonies that helped them to protect from harsh environmental conditions (Choudhari 2015).

It is difficult to study microbial species and their resistant profile worldwide due to their high diversity but the NGS technology along with the metagenomic approach is increasingly growing. Microbial genomics research uses metagenomics as a powerful technique in the extraction and sequencing of DNA directly from a microbial population present in an ecological system (Simon et al. 2009). Metagenomics provides knowledge of microbial diversity and the functional ability of microbes living at different locations such as soil, human intestine, seas, and snow, etc. It also helped to assess the biotechnological ability as well as helps to assess the number of genes present, and the microbe’s biochemical and metabolic characteristics (Von Mering et al. 2007). The composition of entire glacial microbial communities can be determined by PCR-based analysis of the 16S rRNA or 18S rRNA gene using directly isolated glacial DNA as the starting material. It is feasible to classify trapped, proliferating, or dead species that are alive, frozen, or concealed through ice, water, or sediment. DNA libraries constructed from glacial DNA allow to identify potentially new biocatalysts and compounds (Liu et al. 2009). Glacial metagenomics research may provide details on the microorganisms in cold and frozen places on Earth.

Cold and low-temperature environments are less explored by scientists which increases their interest due to the possibility of discovering new species. Here, we concentrate on the diversity and concentration of microorganisms in natural environments to examine the form of bacteria present for centuries in the absence of any natural host. The main goal of the study is to unveil the bacterial communities through DNA sequencing and then to analyze their diversity patterns and relationship with environmental factors through the metagenomics study mainly focusing on alpha and beta diversity.

## Materials and methods

### Site selection, sample collection and preparation

**Fig. 1:**
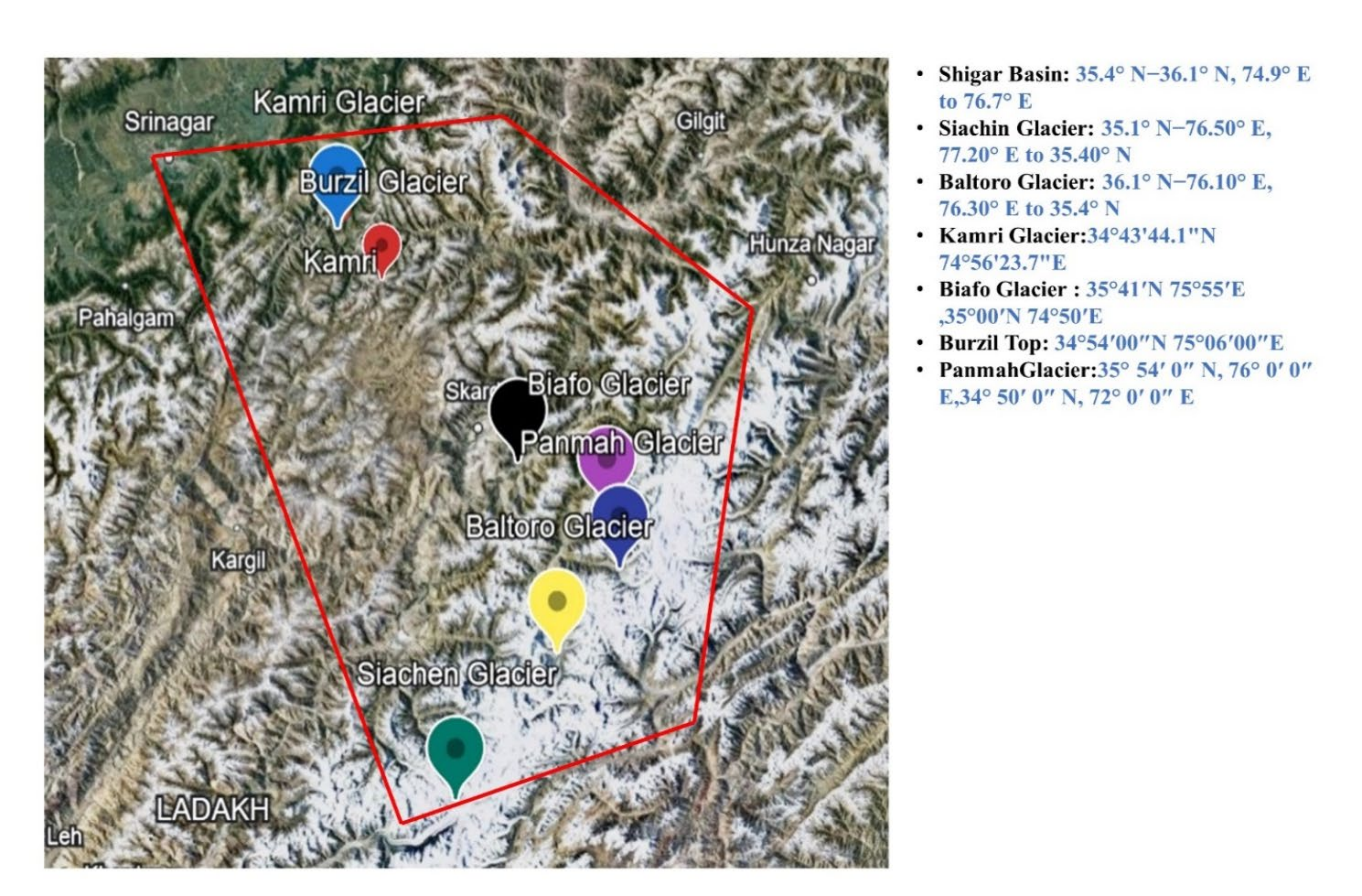
Site map of selected glaciers taken from Google map Kamri, Burzil, Siachin, Baltoro,  Shigar Basin, Biafo, and Panmah

The glacial samples were collected from eight accessible glacier sites from Pakistan i.e. Kamri, Burzil, Siachin, Baltoro, Shigar Basin, Biafo and Panmah. There were about eight samples collected from each sites according to the quadrate method for selection (Fig. 1). Samples were air-dried and grounded to fine powder to reduce heterogeneity and to provide maximum surface area for physiochemical reactions.

### Physicochemical analysis of soil

Different parameters i.e., depth, temperature, pH, EC, sulphate, nitrate, phosphorous, potassium, calcium–magnesium, and pH ice and pH snow respectively, were analyzed to define the physiochemical properties of the samples which were taken from these glaciers. For this purpose, a dried soil sample was digested at 70 °C, filtered, and diluted to 50 ml using deionized water. 70 °C digestion temperature is a commonly used temperature for environmental sample preparation, as it facilitates the dissolution of the sample matrix without causing significant analyte loss. The solution was analyzed for Pb, Cr, Cd, Fe, Na, Cu, Mn, Mg, and K using a flame atomic absorption spectrophotometer. The PerkinElmer AA-7000 Atomic Absorption Spectrophotometer was used to analyze the soil sample content for various elements, including calcium, magnesium, potassium, sulfate, phosphate, nitrate, nitrite, copper, iron, manganese, lead, chromium, and zinc. For water analysis, pH meter was calibrated, and a 50 ml water sample was taken in a flask. Combined electrodes were introduced, and readings were taken after 30 s. Electrical conductivity was measured by calibration of a conductivity meter whose cells were rinsed, and dried and a measured solution was applied. 75 ml of sample was taken, conductivity cells were inserted and readings were taken.

#### Genomic DNA extraction and sequencing

The DNA was extracted using the DNeasy PowerSoil Pro Kit (Qiagen, Hilden, Germany). The integrity purity and conc. of DNA was checked by 1% agarose gel electrophoresis. Using genomic DNA as a template according to the selection of sequencing region-specific primers with barcode and Takara premier Taq version 2.0 (Takara Biotch. Co. Dalian China) were used for amplification by PCR. 16S V4 primers (515f and 806r) were used to identify bacterial diversity. The concentration and length of the PCR product were identified by 1% agarose gel electrophoresis. By comparing the concentration of PCR products by gene tools analysis software the volume of each sample was calculated with respect to the principle of equal quality and then the PCR products were mixed the E.Z.N.A PCR Gel Extraction Kit was used to recover PCR mixed products. The length of the main band was within the normal range (for example, 16S v4:290–310 bp/16S v4–v5:400–450 bp, etc.) and can be used for further experiments. The database was built according to the standard process of nebnext ultra-DNA library prep kit for Illumina (New England Biolabs USA). The amplified library was sequenced by PE250 using Illumina Nova 6000 platform (Guangdong Magigene Biotechnology Co. Ltd. Guangzhou China). Data was submitted at NCBI with following IDs Project-16S Glacial project SRA. PRJNA997782-ID: 997782-G1-G8 (SRX21348208-SRX21348215.

### Sequencing data processing

Fastp (an ultra-fast all in one fastq preprocessor version 0.14.1 https://github.com/opengene/fastp) was used to cut the sliding window quality (-w4-m20) of two end raw reads data respectively and use cut adapt software (https://github.com/marcelm/cutadapt/) to remove the primer information at both ends of the sequence. For the data of two terminal sequencing according to the overlap relationship between PE reads usearch-fastq’mergepairs were used to filter the inconsistent tags and obtain the original one’s raw tags.

### Metagenomics analysis workflow

Open-source Bioconductor project and some CRAN packages were used for data analysis.

Filter and Trim: Low-quality sequencing reads and trimming of the reads was done to a consistent length. The different trends of decreasing or increasing average quality in sequencing reads were analysed.

Infer Sequence Variants: After filtering, by the typical amplicon bioinformatics workflow, clustering of the sequencing reads into operational taxonomic units (OTUs) was done. Groups of sequencing read were made based on differences by less than a fixed dissimilarity threshold. High-resolution DADA2 method was used to infer amplicon sequence variants (ASVs) exactly, without imposing any arbitrary threshold, and thereby resolving variants that differed by as little as one nucleotide.

Dereplication: Dereplication substantially reduced computation time by eliminating redundant comparisons. The DADA2 method relied on a parameterized model of substitution errors to distinguish sequencing errors from real biological variation. Intensive parameter learning was done computationally with multiple iterations of the sequence inference algorithm for estimation of the error rates from a (sufficiently large) subset of the data. It removed all substitution an indel errors from the data.

Construct Sequence Table and remove chimeras: Higher-resolution analogue of the common “OTU table was made by the DADA2 method produced. Inferred forward and reverse sequences were mixed and non-overlapping reads were removed.

Assign Taxonomy: The DADA2 package was used by implementing the naive Bayesian classifier method for the purpose of assigning taxonomy to the sequence variants.

Taxonomic Filtering: Data was filtered and agglomerated taxa for effective analysis of microbiome count.

Prevalence Filtering: Featured prevalence of the dataset was made based on appearance of taxon at least once.

Agglomerate Taxa: Functional redundancy in the microbial community, was found by agglomeration of the closely related taxa.

Calculating abundance value before and after transformation: Proportions and relative abundances were calculated.

## Results

Study areas Kamri, Burzil, Siachin, Baltoro, Shigar Basin, Biafo and Panmah are prominent sub-basin of Upper Indus Basin (UIB), Pakistan (Fig. 1). Geologically, they occupy partly southern margin of Karakoram plate, Hunza Basin, Shyok Basin, and partly on the northern margin of Kohistan in the Northern, Southern and western basin. Altitude ranges from 2775 to 8611 m. Almost 50% of the glacier area is covered by 4 major glaciers and among those of Baltoro (607 km^2^), Biafo (406 km^2^), Shigar Basin (358 km^2^), and Panmah (274 km^2^) are also included.

## Physicochemical analysis

The physiochemical features of the glacier samples which are depth, temperature, pH, EC, Sulphate, nitrate, phosphorous, Potassium, Calcium–Magnesium, and pH ice and pH snow respectively, have been taken to define the physiochemical parameters, which were represented in the following Table 1.

**Table 1:** Physicochemical parameters of glaciers samples

Sample	G1 Control	G2 Kamri top	G3 Burzil top	G4 Siachin	G5 Baltoro	G6 Shigar basin	G7 Biafo	G8 Panmah
Depth (cm)	302	262	264	300	158	140	120	130
Temp. (°C)	5	− 4	− 20	− 5	− 10	− 20	−20	− 20
pH	7.37	7.62	8.23	6.49	7.21	6.23	6.12	6.91
EC uS/cm	114 + 0.03	242 + 0.02	349 + 0.05	349 + 0.05	437 + 0.03	67 + 0.03	730 + 0.01	730 + 0.01
S %	0.42	0.11	0.04	0.04	0.01	0.05	0.15	0.05
Nitrates %	0.29	0.02	0.09	0.05	0.08	0.06	0.12	0.10
P %	0.03	0.01	0.03	0.02	0.07	0.04	0.09	0.08
K mg/kg	440 + 0.03	144 + 0.02	206 + 0.06	244 + 0.02	220 + 0.03	200 + 0.05	264 + 0.03	284 + 0.01
Ca-Mg %	3.67	4.21	4.33	5.33	4.67	5.41	5.67	5.77

From the Table 1, it was noted that temperature in winter was lowest in Biafo (− 20 °C), whereas it was maximum − 4 °C in the Kamri top. The pH of Burzil top was maximum (8.23), whereas the pH of Biafo was minimum (6.12). Electrical conductivity which was indicator of redox potential and metabolic activity too was highest in Biafo and Panmah. The nutrients concentration found highest in the Biafo where EC was also highest. Remaining sites showed less variability in the levels of nutrients present. Nutrient concentration as well as EC was considered as an indicator for prospect high diversity of bacteria in the sites. High temperature was considered to be indicator for hosting unusual bacterial species which were not representative organisms of glaciers.

The table also reveals seasonal variations in glacier physicochemical properties, with summer samples having shallower depths, lower temperatures, and slightly acidic pH values, while winter samples have higher electrical conductivity, sulfur content, nitrates, phosphorus, potassium, and calcium-magnesium content.

### Alpha diversity analysis

**Fig. 2:**
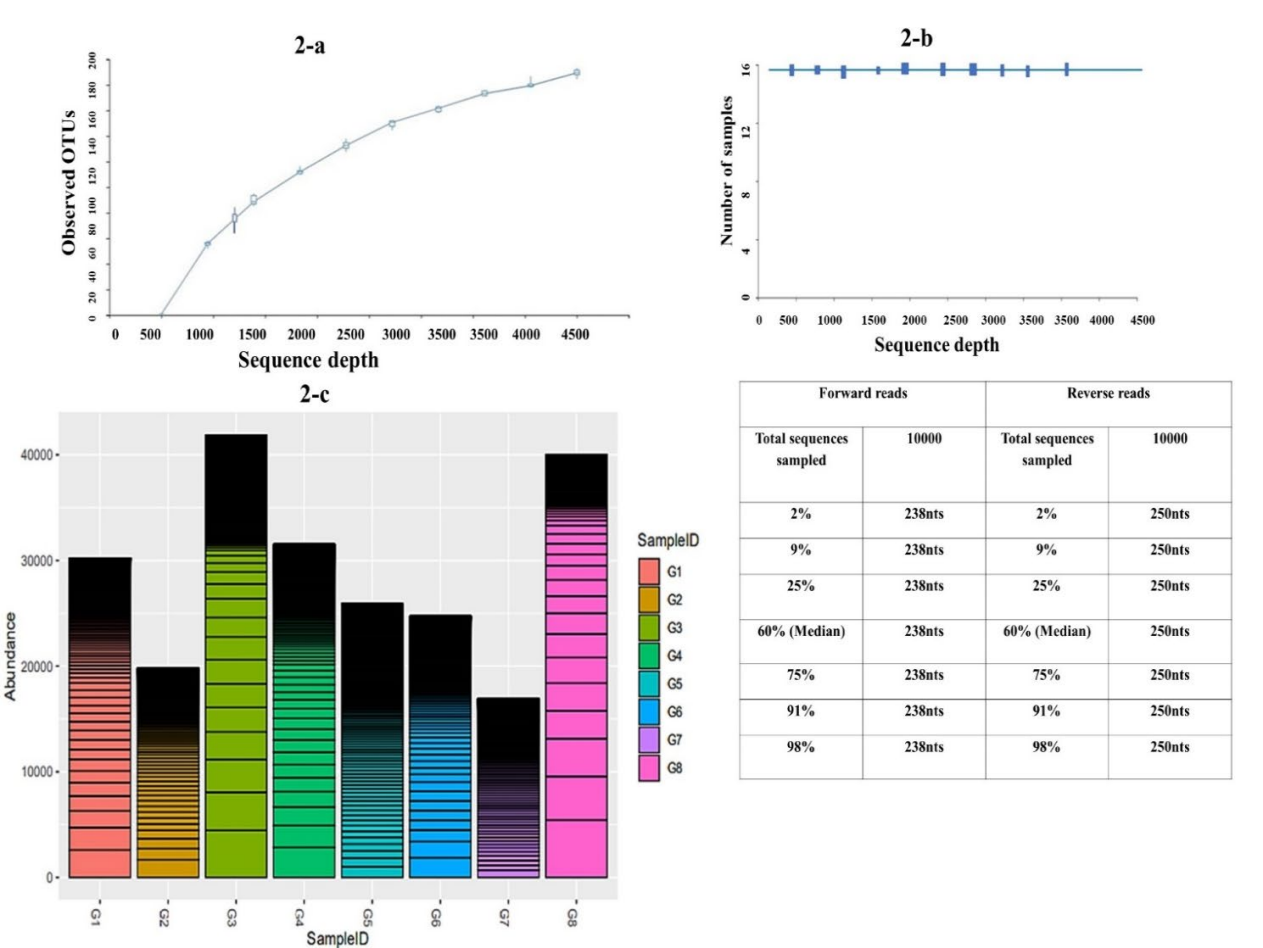
**a** shows the data obtained after demultiplexing sequences and parametric Box plot applied on the sequencing depth (parameter) on the samples, forward and reverse reads of the sequence obtained from the glacier samples, **b** The soil of the glaciers was tested and in forward reads the graph was generated using random sampling of 10,000 out of 695,238 sequences without replacement. The minimum sequence length identified during subsampling was 238 bases. Outliers are ignored for better results and symmetry. graph was generated using random sampling of 10,000 out of 695,238 sequences without replacement, **c**  OUT’s abundance was plotted in each sample, among all glacial samples G3 showed maximum OTU abundance and G7 showed minimum abundance.

Raw data was subjected to quality assessment after trimming and truncation of low-quality reads. Trimming parameters were combined with standard filtering parameters, the most important being the enforcement of a maximum of 2 expected errors per reads (Fig. S1). After excluding non-quality reads all samples from G1 to G8 had more than 80,000 reads. Demultiplexed sequence was applied on the sequence forward reads as well as reverse reads and it observed that the minimum sequence count for forward read was 238nts, in forward reads its median was 238nt, and in reverse reads it was 250nts respectively. Per sample sequence counts of 10 samples showed that a higher number of sequence counts was observed in reverse reads sample (Fig. 2).

The minimum sequence length identified during subsampling was 250 bases. The median was minimally influenced by outliers than the mean, and was generally the chosen central tendency indicator when the distribution was not symmetrical. Demultiplexing was the step involved in processing the information in order to know which sequences came from which samples after they had all are sequenced together. By applying the box plot on the obtained result on the samples taken from the 7 glaciers and one control, it was observed that the given samples had the regular linear increase in observed Phylogenetic diversity with increase in sequence depth, which means higher or larger the sequence depth more and greater the similarities (Fig. 2). Alpha diversity was analyzed to check the richness and evenness in different taxa in the seven glaciers. Operational taxonomic units were used to classify groups of closely related individuals against the sequence depth. Isotherm appeared when sequence depth was measured against each sample. Figure 2a is the isotherm which means there was a linear increase in observed OTUs with an increase in sequence depth, which means the higher or larger the sequence depth the greater the similarities. It is the regular linear increase in observed Phylogenetic diversity with the increase in sequence depth, which means the higher or larger the sequence depth, greater the similarities.

**Fig. 3:**
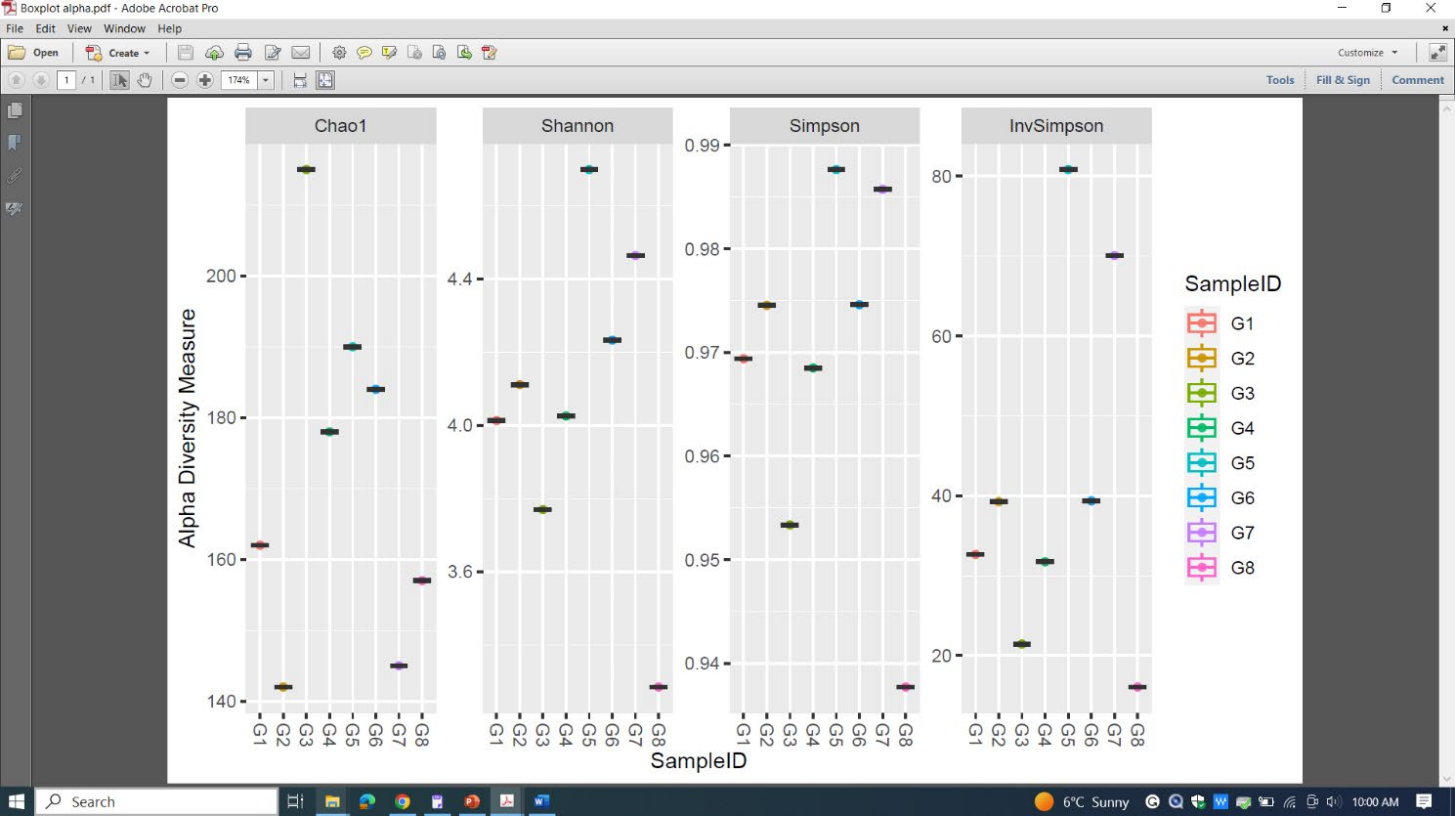
Alpha diversity with reference to Chao1, Shannon, Simpson, and Inverse Simpson indexes. The Shannon index is in the range of 2.2 to 2.6. Generally, its value is between 1.5 and 3.5 in most ecological studies. The Shannon index rises as the community's richness as well as its evenness rises. This describes the index for a species' Beta diversity in a population since it is the combination of all the pairs collected from the various samples.

The alpha diversity of glacier samples labeled G1 to G8 was measured by Chao1, Shannon, Simpson, and Inverse Simpson metrics (Fig. 3). These metrics provided different perspectives on alpha diversity. Chao1 estimated total species richness, while the Shannon and Simpson indices provided information about the distribution of species and their relative abundances. The Inverse Simpson index also considered richness and evenness but placed more emphasis on rare species. By analyzing these metrics for these glacier samples (G1–G8), we got insights into the diversity, richness, evenness, and dominance patterns within each sample. This information gave us valuable for understanding for the ecological health and dynamics of glacier ecosystems.

The y-axis values ranged from 140 to 200 represented the Chao1 values for each glacier sample. A higher Chao1 value indicated a higher estimated species richness in G3 sample. It indicates that G3 likely had a higher estimated species richness compared to other samples. Dots of G4, G5 and G6 were relatively close in height, it suggested that the estimated species richness was similar across these samples. On the other hand, there was a significant difference in dot heights for G1, G2, G7 and G8 indicating variations in species richness between these samples.

By observing the heights of the dots for Shannon values G5 suggested a more diverse community with a more even distribution of species with a Shannon value of 4.6. The dots of G3, G4, G6 and G7 were relatively close in height, which suggested that the diversity and evenness of species were similar across these samples. G1 was closer to G2 but there was a significant difference in values of G1 and G8, it indicated variations in species diversity and relative abundances between samples of those two glaciers. G8 and G3 showed a decreased value of the Shannon index which might be due to environmental stress.

The Simpson index measured the dominance or concentration of species in a community. It calculated the probability that two randomly selected individuals from the community belonged to the same species. A higher Simpson value indicated a lesser diverse community with a higher dominance of one or a few species. G5 and G7 had higher dots with a Simpson index greater than 0.92, which indicated that they had a higher dominance and concentration of species compared to G8 whose value was below 0.94 and G8 between 0.95 and 0.96. The Shannon index value of G1, G2, G4 and G6 were intermediate between 0.96 and 0.98 suggesting that the dominance and concentration of species were similar across those samples.

The Inverse Simpson index was the reciprocal of the Simpson index. It gave more weight to rare species, emphasizing their contribution to diversity. It quantified the effective number of equally abundant species. The y-axis values ranged from 20 to 80 represented the Inverse Simpson diversity values for each glacier sample. G5 and G7 had higher index values between 60 and 80 as compared to G3 and G8 whose values were below 20, which indicated that G5 and G7 had a higher richness and evenness of species, with a greater contribution from rare species. The dots of G1, G2, G4 and G6 were relatively close in height, which suggested that the richness and evenness of species, considering rare species, were similar across those samples. On the other hand, there was a significant difference in dot heights of G5 and G8, thus indicating variations in diversity, with more emphasis on rare species, between those samples. Overall G3 and G5 showed increased values of alpha diversity matrices thus showing an increase in diversity, richness, evenness, and dominance patterns. So overall highest abundance and diversity was seen in Siachin in summer and Kamri in winter.

**Fig. 4:**
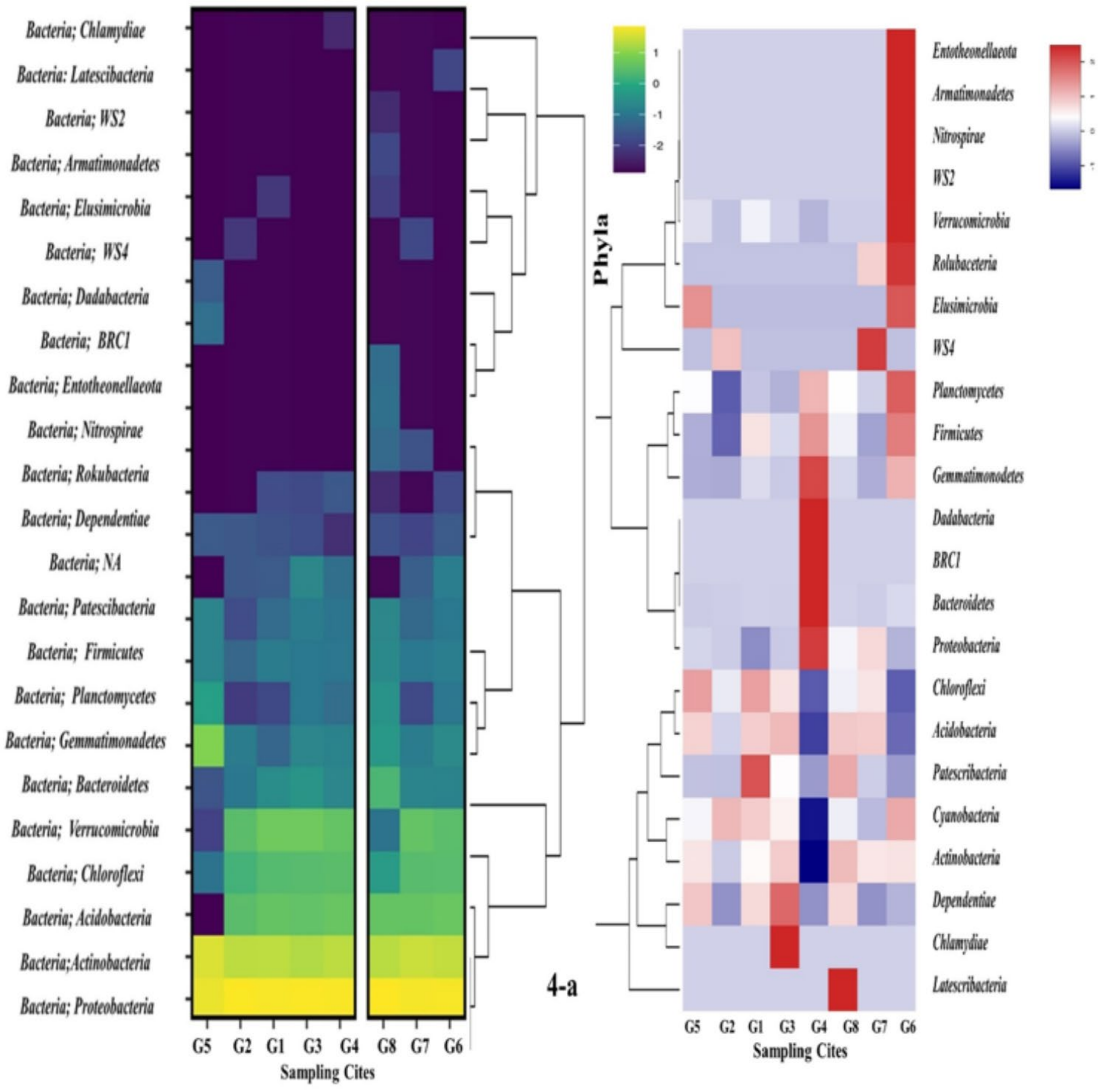

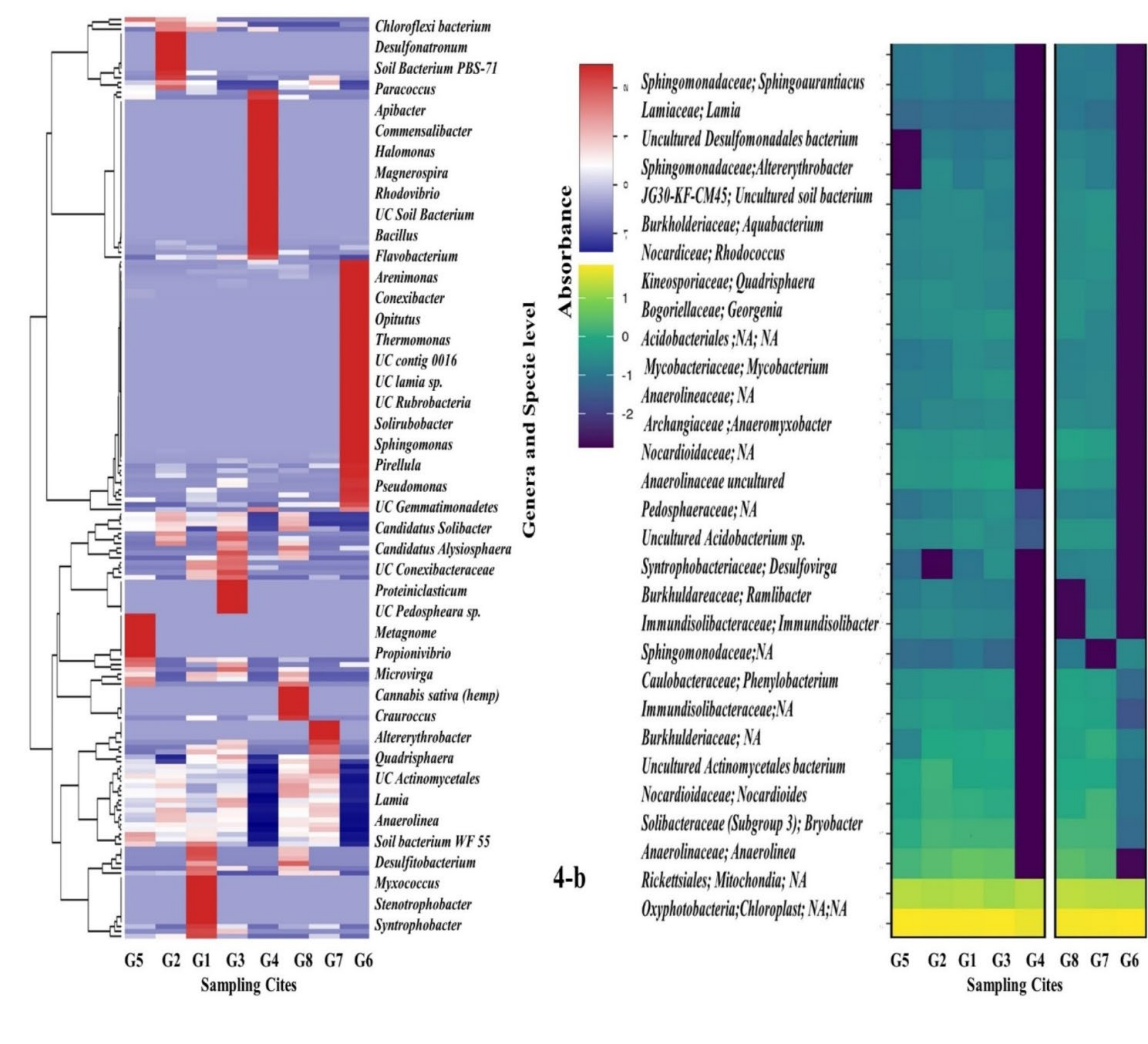
**a, b**  Heat maps showing the distribution of taxa at phylum, genus and species level. UC stands for uncultured OTUs.  NA stands for those OTUs which have not been classified yet. Values are the taxa's relative abundance in the percentage of total sequences and the figure shows taxa with an abundance of > 0.01 percent. All values are rounded to one digit; thus, the abundance in one sample of a taxon with a value of 0.0 lies between 0.00 and 0.04 percent. Different algorithms used gave slightly different results

Phyla *Proteobacteria* displayed higher OTUs accompanied by *Actinobacteria*,* Firmicutes*, and then *Bacteriodetes* in the heat map correlated with the relative percentage of each OTU in the samples. The *Proteobacteria* had the highest diversity in all the selected glaciers (Fig. 4a, b). Relative abundance in the percentage of total sequences at abundance of 0.01% presented distinct dominant phyla and specie in all samples. Distribution of OTUs were the taxa ’s relative abundance in the percentage of total sequences and the figure showed taxa with an abundance of 0.01 to 0.04%.

### Beta diversity analysis

**Fig. 5:**
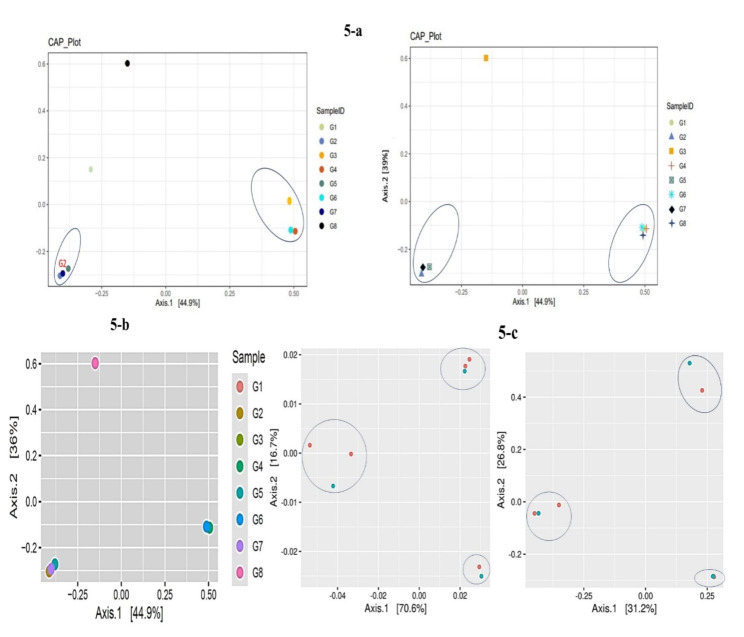
**a** AP plot to visualize species distribution without aesthetics and with aesthetics added. **b** CA1 and CA2 with 44.9 coordinates at x and 36.0 at y-axis showed that control sample is lying differently as compared to all glacial sites. **c** Unifrac and unwunifrac analysis showed that with the distribution of samples as two subjects based on physichochemical parameters similarities and similarities in OTUs, both subjects again ended up in sharing same clusters

Beta diversity analysis was used to measure and understand the variation in species composition between different samples within an ecosystem. Cumulative abundance of species along a gradient or ordination axis was estimated by CAP plots (Fig. 5a) which allowed us to visualize patterns of species distribution. In the above Fig. 5a CAP plot with and without aesthetics added was shown which referred to comparing two versions of a Cumulative Abundance Profile plot, one with basic representation and another with visual enhancements to better visualize and interpret patterns of species composition among different samples. The percentages on the x and y-axis indicated the proportion of explained variation for each axis.

In Fig. 5b G1 was located farther to both axis. G2, G5, G1 and G7 were located in clusters indicated more similarity in species composition. Similarly, G4, G3 and G6 followed the same pattern. The greater distance of G1 from other samples showed its species dissimilarity from other glacier samples. In Fig. 5c samples were distributed in two subjects based on similarities in alpha diversity indices and shared OTUs. Both subjects were shared by all clusters which showed that variations among samples were not significant.

**Fig. 6:**
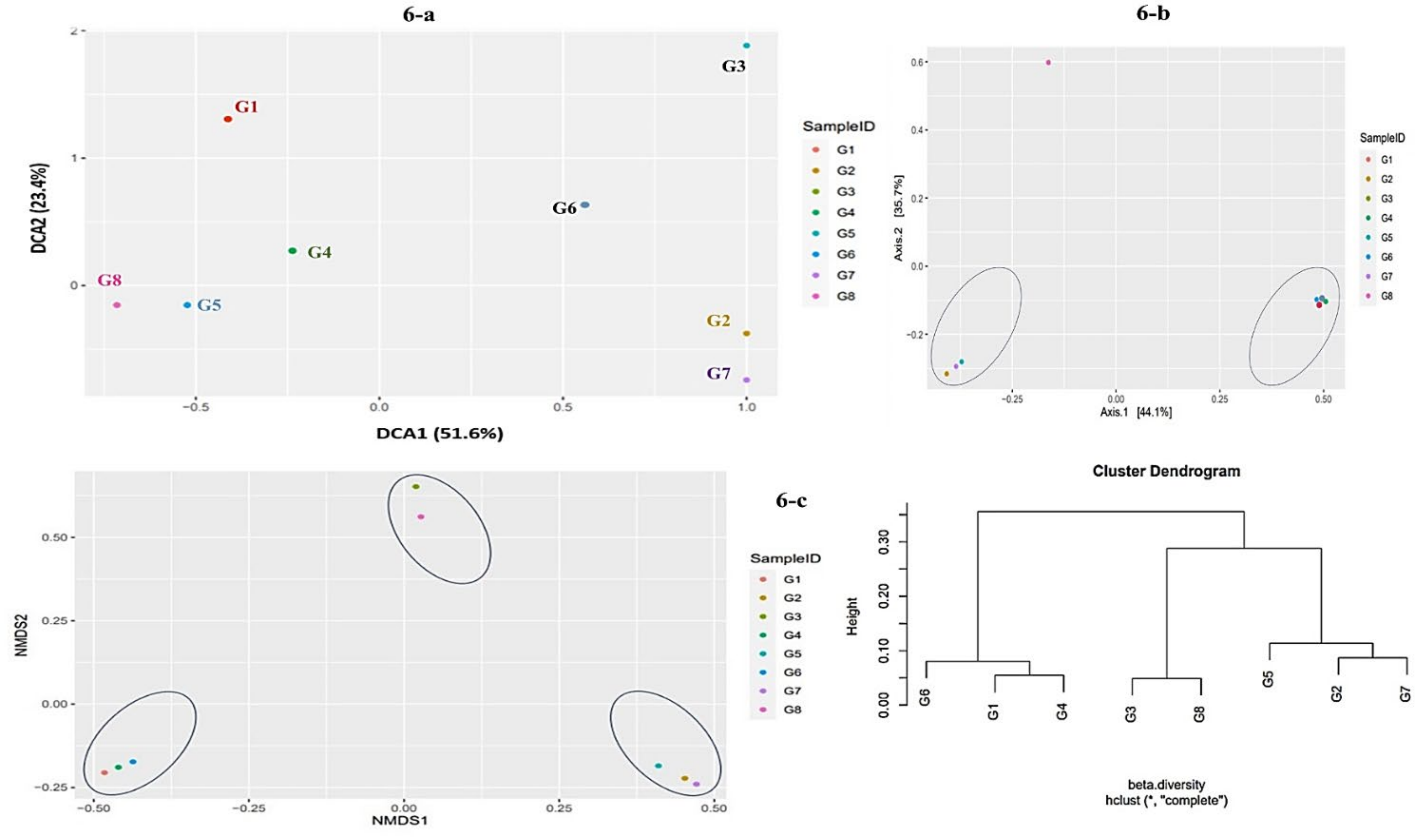
**a** DCA plot at DCA1 with 51.6% coordinates and DCA2 with 23.4% coordinates, **b** CCA plot with clustering of glacial samples into two distinct groups**c** NMDS plot at NMDS1 and NMDS2 axis and Beta cluster hclust in the form of cluster dendrogram

DCA was often applied as an initial step before choosing the appropriate ordination method. It was used to reveal the underlying structure in multivariate species abundance data (Fig. 6a). In the graph layout, the x-axis was labeled as DCA1 (51.6%) and the y-axis was labeled as DCA2 (23.4%). The graphs showed that dots were dispersed across the plot, which suggested a gradient in species composition and environmental factors. The G1 and G6 were present close along the y-axis while G7 and G2 were present close along the x-axis. It indicated similarity in their species composition and low scores on that axis. G5 was located farther away from the origin having high scores and showed dissimilarity from other samples composition. It showed that the species composition of kamri was less similar to other glacier samples. CCA plot was used to analyze and model the relationship between species abundance data and environmental variables (Fig. 6b). Different percentages on the x and y-axis indicated the proportion of variance for each axis. G1 being control clearly showed significant variation as compared to all other samples. Glacial samples clustered in two groups with G7, G6 and G2 being in one cluster and G3, G4, G5 and G8 in other cluster.

Interpreting a NMDS plot involved understanding the arrangement of samples (dots) in the reduced-dimensional space represented by NMSD1 and NMSD2 axes (Fig. 6c). The positions of the dots provided insights into the similarity or dissimilarity of glacier species composition. In the above graph G1, G4 and G6 dots were closer to each other on the NMDS plot, which showed similarity in their species composition. G3 and G8 were also closer to each other. Similarly, G5, G2, G7 also followed the same pattern and depicted similar environmental conditions. Hierarchical cluster was used to identify patterns of community similarity and dissimilarity, which could have ecological implications and helped in the interpretation of species distribution across different environments or conditions. In the above Fig. 6c, the cluster dendrogram showed five clusters from top to bottom. There was a close similarity between G1 and G4. Glacier samples G3 and G8 had less distance and more similarity index. The same pattern was followed by G2 and G7. By moving from bottom to up more species added to form a new cluster i.e., G6, G1 and G4 showed similar species. Similarly, G2, G5 and G7 joined to form a cluster. At the top of the dendrogram all samples combined to form a single cluster. The reading scale showed that as more clusters joined, distance of vertical lines increased depicting less similarity index between species. For our understanding, we did cut the dendrogram at 0.10 reading to consider only those clusters that were more closely related.

PCoA on the basis of Bray–Curtis dissimilarity was performed by using the Bray–Curtis dissimilarity metric to quantify how different the species compositions were between pairs of samples. Then, we applied PCoA to these dissimilarity values to create a lower-dimensional representation of samples that preserved their pairwise dissimilarity relationships.

**Fig. 7:**
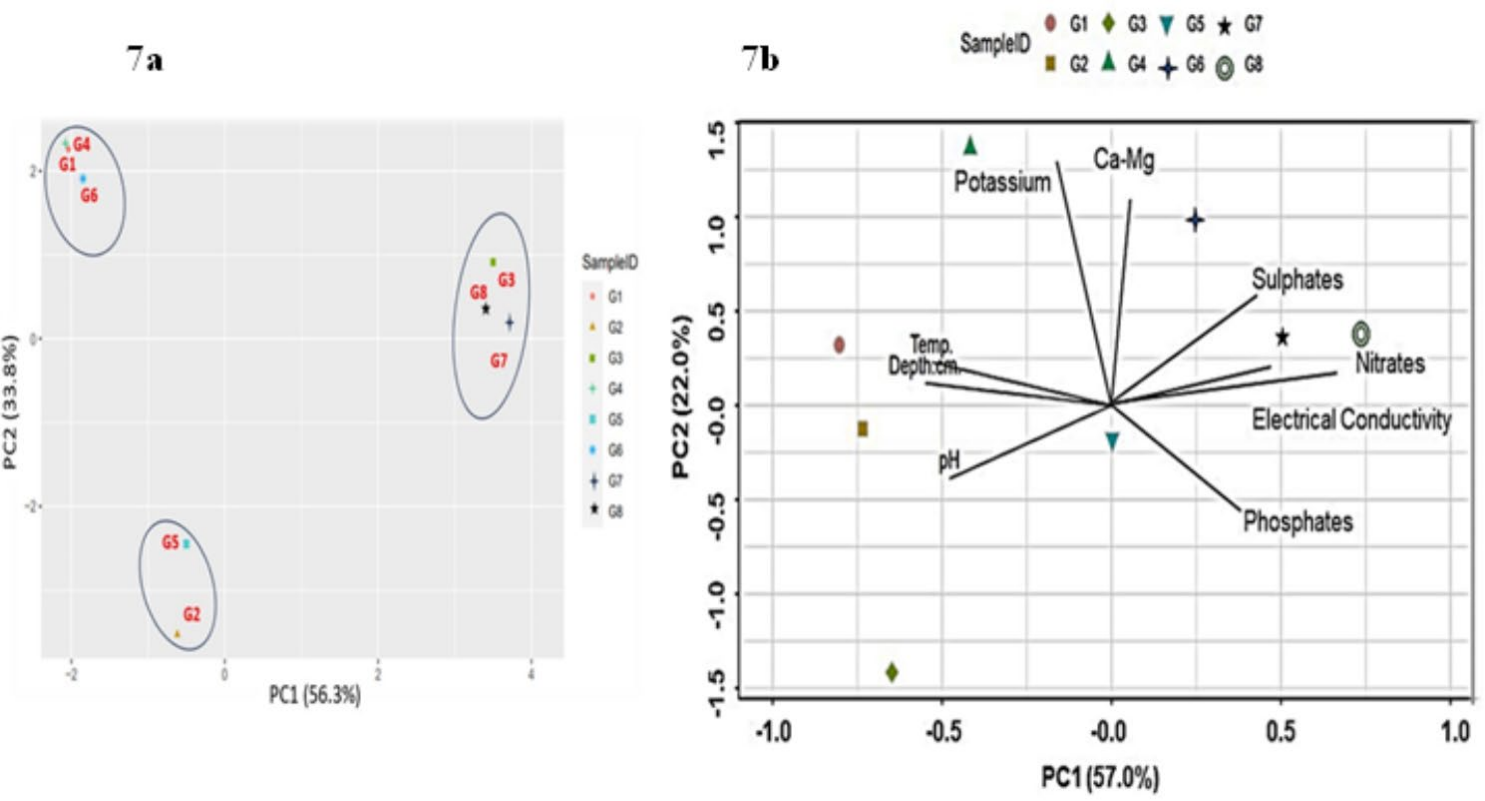
**a** PCoA plot on the basis of Bray–Curtis dissimilarity at axis 1 with 56.3 % coordinates and at axis 2 with 33.8% coordinates, **b** RDA:CCA plot at PC1 with 57.0% coordinates and at PC2 with 22.0% coordinates

In Fig. 7a graph was plotted at axis1 with 56.3% coordinates and at axis 2 with 33.8% coordinates. The different axes of PCoA accounted for different variations. The result was in the form of two clusters based on phylogenetic distance metrics. PCoA focused on distance or dissimilarity matrices rather than raw data. The result was in the form of two clusters based upon phylogenetic distance metrics. G6, G1 and G4 were present in the form of clusters but at a distance from other glacier samples thus indicating greater pairwise distances between those samples. The G3, G7 and G8 were also clustered together indicating these groups of samples were more closely similar to each other than others. G2 and G5 were clustered together too. The clustering of dots on the graph also reflected species turnover which measured the change in species composition between different sites or conditions. Statistical tests, PERMANOVA helped to determine that observed clustering was statistically significant. RDA: CCA plot was a graphical representation to visualize the relationships between glacier samples and environmental variables. The above plots combined the strengths of both RDA and CCA to explore how environmental factors influenced the composition of species within samples. In the given figure factor datasets i.e., temperature, depth, Electrical conductivity, Nitrates, Phosphates, Sulphates, Ca–Mg, Potassium and pH were used to evaluate their effect on glacier samples (Fig. 7b). By looking at the graph it was observed that other than G3 all other sites OUT’s were directly impacted and influenced by the relevant physichochemical parameters suggesting strong redox potential and active metabolic activity at respective sites.

## Discussion

Research on microbial communities in extreme environments is one of the most exciting fields in microbiology. Microorganisms that make up these communities are able to withstand extreme temperatures, pressure, oxygen levels, and salinity. It is crucial to investigate these societies because it sheds light on the boundaries of Earthly life and the possibility of extraterrestrial life. Glaciers are among the harsh environments that harbor a variety of extremophiles (Hauptmann et al. 2014). There are certain adaptations microorganisms have made to cope with the harsh conditions of glaciers, such as the ability to synthesize antifreeze proteins and the ability to break down complex chemical molecules. As a result of these modifications, microbes can now thrive in conditions that would normally be uninhabitable. In addition to providing valuable insight into extremeophile adaptation, examining microbial communities within glaciers may also offer biotechnological applications. The vast number of glaciers in Pakistan underscores their importance as a source of freshwater for rivers, thereby playing a crucial role in sustaining ecosystems and economies (Hauptmann et al. 2014). The results obtained from the diverse analyses shed light on the complex microbial communities thriving in these extreme environments. Glaciers, formed from the accumulation of snow over time, host a unique range of microorganisms that have adapted to the challenging cold and nutrient-scarce conditions.

The surroundings of the seven glacier study areas were almost similar. We investigated snow sample bacterial communities from selected glaciers and identified OTUs, which were mostly associated with *Burkholderiales* (*Proteobacteria*), *Xanthomonadales* (*Proteobacteria*), *Cytophagales* (*Bacteroidetes*) and *Bacillales* (*Firmicutes*) (Cameron et al. 2015; Michaud et al. 2014). For understanding, bacterial diversity from the selected seven sites of the glaciers was used as an indicator for the anthropological impact on the natural environment. Approximately 40% of the five detected bacterial groups were found in most glaciers, particularly *Proteobacteria*, *Rhoddococcus*, *Firmicute*,* Bacteriodetes*,* Actinobacteria* clusters. In Xinag et al. (2009a, b) reported that *Proteobacteria*, *Cyanobacteria*,* Firmicutes*, and *Bacteroidetes* were the most commonly found phyla in glaciers (Xiang et al. 2009a, b). The dominancy of *Proteobacteria* cluster was dominant in all the phylum of bacteria, the highest number of species of *Proteobacteria* was in Burzil. *Actinobacteria* in baltoro and most species of Firmicutes in the siachin. The least number of species were found in the Kamri, this showed that *Proteobacteria* could easily be survived in the harsh cold environment they had the genome variability to survive the growth.

Alpha diversity and microbial community composition provided insights into the richness, evenness, and diversity of bacterial species within each glacier sample. The observed shifts in alpha diversity metrics, such as Chao1, Shannon, Simpson, and Inverse Simpson indices, across the different glacier samples indicated variations in species composition and distribution. For instance, the higher Chao1 values in certain samples, like siachin glacier suggested a potentially richer species diversity, while other metrics showed abundance in kamri and siachin. The differences in dominance patterns, as indicated by the Simpson index, further emphasized the distinct bacterial communities within each glacier. These findings highlight the adaptability and resilience of bacterial populations in response to the unique environmental conditions of each glacier.

This study’s findings carried both microbiological and ecological significance. Through the exploration of bacterial diversity in different Pakistani glaciers using metagenomic techniques, as well as the analysis of alpha and beta diversity using diverse tools, we had gained valuable insights into the intricate microbial communities thriving in these harsh environments. The adaptability and resilience of glacier bacteria, particularly in the face of extreme conditions, offered valuable insights into extremophile physiology and potential applications in biotechnology. The remote pristine environments, with minimal or none human interruption have been reservoirs of paleanbacteria, and hosting unique and ignored genomic pool. Revived bacteria could be an indicator of uncoming epidemics, and antibiotic resistance genes. Our samples G4 (Siachin) and G6 (Shigar Basin) showed prevelance of genera *Lamia*,* Mycobacterium*,* Pseudomonas* which were reported to be sources of multidrug resistance and infections. At specie level a public health threat genera *Chlamydiae* was observed in highest OTUs. According to Sachse and Borel (2020) it could be a red flag for upcoming epidemics especially veterinary related epidemics. Almost 14 species of *Chlamydiae* were reported to be the sources of epidemics in life stock and zoonotic infections. The study also highlights the potential hazards of reviving ancient microorganisms from glaciers due to climate change and seasonal variations. These dormant microorganisms, with unknown pathogenic potential, could pose new public health risks. The study also highlights the diversity of bacteria, particularly those with multidrug resistance genes, and the potential for new epidemics. Understanding these communities is crucial for predicting and mitigating risks.

In the realm of metagenomics, this study contributed to a growing body of knowledge that bridges the gap between microbial life and environmental conditions. Future research could extend these insights to explore specific metabolic pathways, functional roles of individual microbial taxa, and potential biotechnological applications harnessed from these cold-adapted organisms.

## Supplementary Information

Supplementary Material 1.

## Data Availability

Data is available at NCBI with following IDs Project-16S Glacial project SRA. PRJNA997782-ID: 997782-G1-G8 (SRX21348208-SRX21348215).
