# Supplementary material for: Resurrected microorganisms: a plethora of resting bacteria underway for human interaction
Source: AMB Express. 2024 Sep 28;14:106. doi: 10.1186/s13568-024-01750-z (PMC11438741; doi:10.1186/s13568-024-01750-z)
Supplement: Supplementary file 1 — Supplementary Material 1. [file 13568_2024_1750_MOESM1_ESM.docx]

**Resurrected Microorganisms: a plethora of resting bacteria underway for human interaction**

**Supplementary Material**


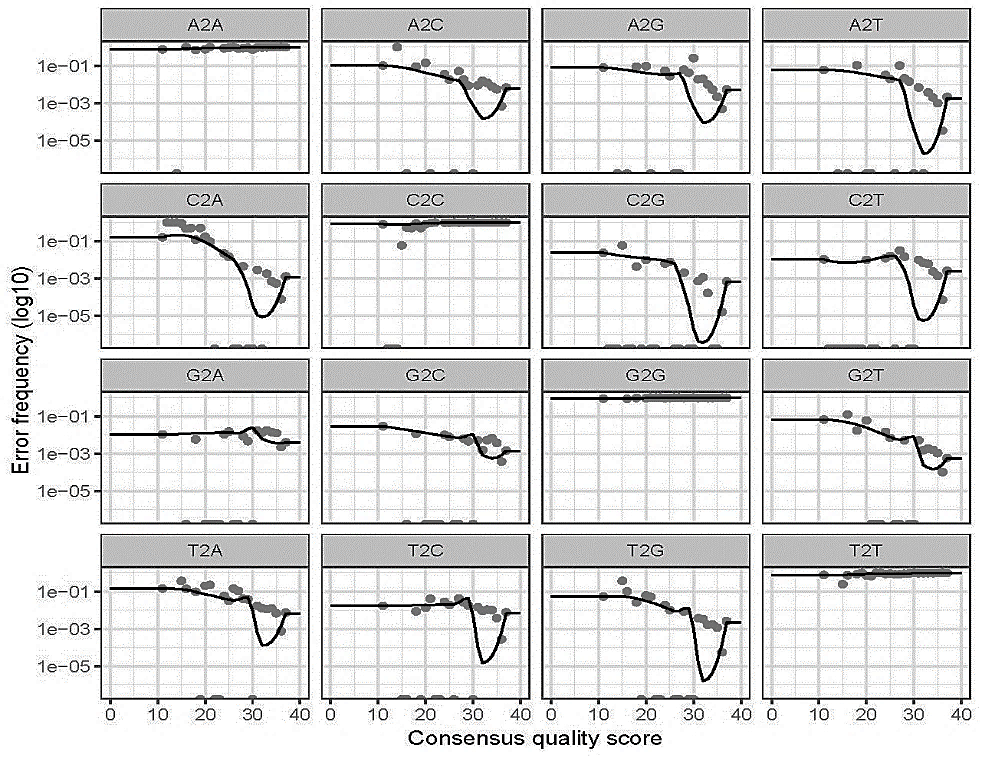

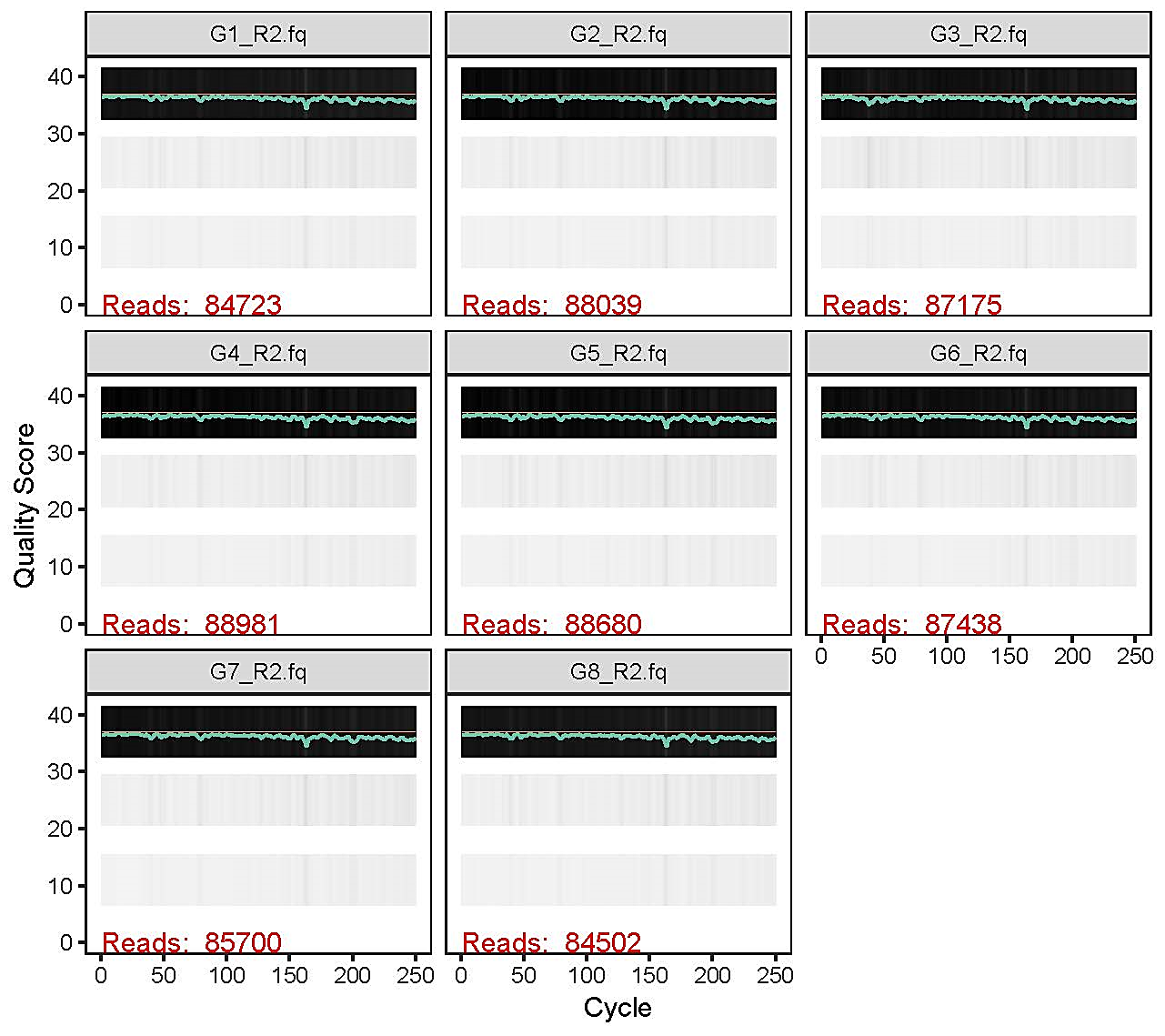


**Fig. S1**(a-b): a) Quality score of G1-G8. b) Reads per sample. Average number of reads in all samples were more than 84,000 reads. Error frequency among ACTG was also estimated to be at acceptable level.
